# Supplementary material for: Recent Advances in SARS-CoV-2 Main Protease Inhibitors: From Nirmatrelvir to Future Perspectives
Source: Biomolecules. 2023 Sep 2;13(9):1339. doi: 10.3390/biom13091339 (PMC10647625; doi:10.3390/biom13091339)
Supplement: Supplementary file 1 [file biomolecules-13-01339-s001.zip › biomolecules-2558099-supplementary.pdf]

Supplementary Material for:

Recent Advances in SARS-CoV-2 Main Protease Inhibitors: From Nirmatrelvir to Future Perspectives

Table S1. Most relevant SARS-CoV-2 M^{pro} inhibitors discovered so far.

Compound	Chemical class	Structure	IC_{50} (nM)[a]	Mechanism	EC_{50} (μM)[c]	Discovery method	References
**1 NIRMATRELVIR**	**Nitriles**	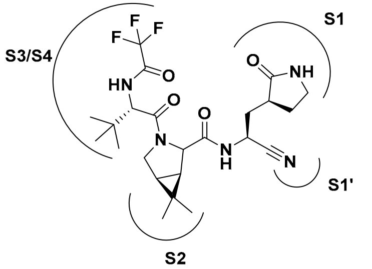	**3.11[b]**	**Covalent Reversible**	**0.075**	**Lead optimization**	[1]
**2 PF-00835231**	**Hydroxy ketones**	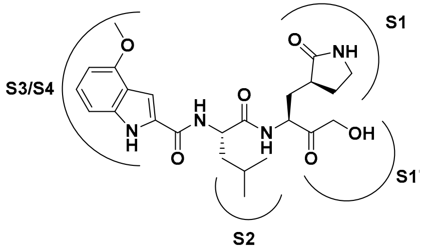	**4[b]**	**Covalent Reversible**	**0.23**	**Drug repurposing**	[1]
**3 PF-07304814**	**Hydroxy ketones (phosphate prodrug)**	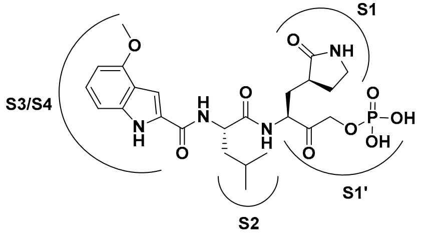	**27.9[b]**	**Covalent Reversible**	**1.4**	**Drug repurposing**	[1]

4	Nitriles	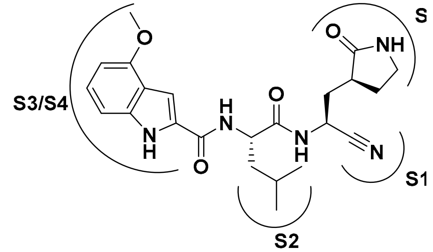	27.7b	Covalent Reversible	1.4	Lead optimization	[1]
5	Ketones	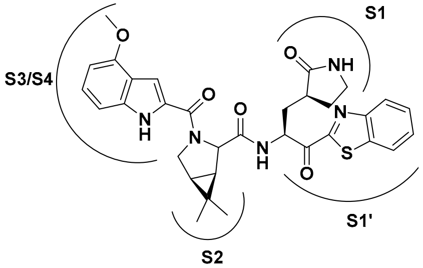	230b	Covalent Reversible	5.6	Lead optimization	[1]
6	Ketones	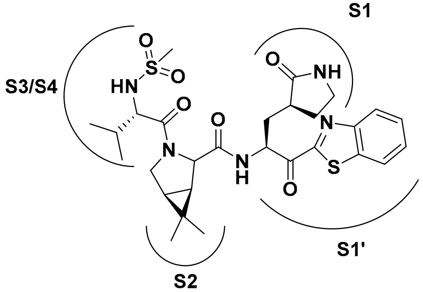	7.93b	Covalent Reversible	0.9	Lead optimization	[1]
7	Ketones	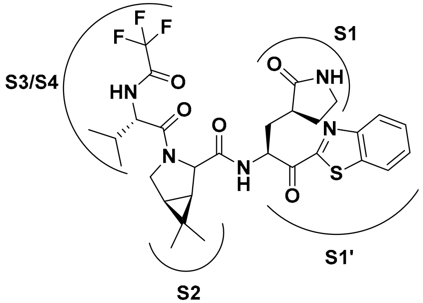	12.1b	Covalent Reversible	0.85	Lead optimization	[1]

8	Nitriles	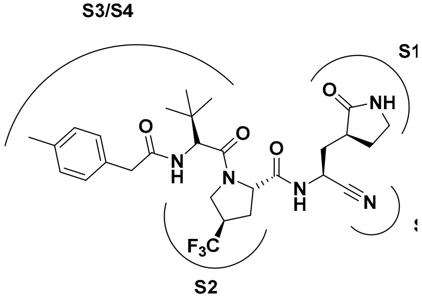	4[b]	Covalent Reversible	0.019	Lead optimization	[2]
9	Nitriles	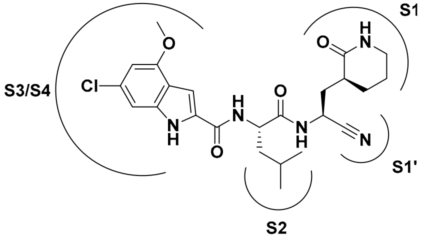	9[b]	Covalent Reversible	2.2	Lead optimization	[3]
10	Nitriles	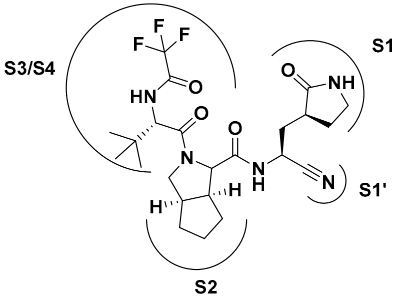	18	Covalent Reversible	0.31	Lead optimization	[4]
11	Nitriles	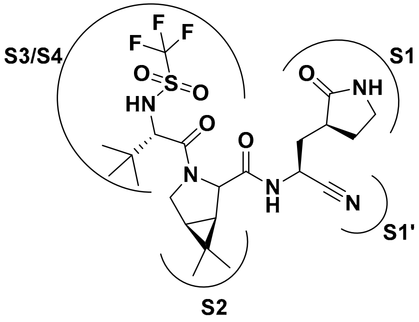	22	Covalent Reversible	0.17	Lead optimization	[4]

12<br>BBH-2	Nitriles	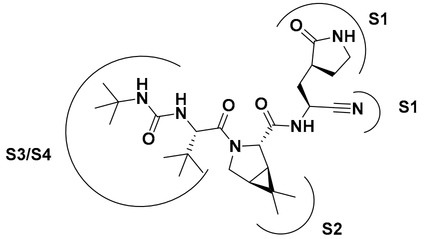	26[d]	Covalent<br>Reversible	0.88	Lead<br>optimization	[5]
13<br>NBH-2	Nitriles	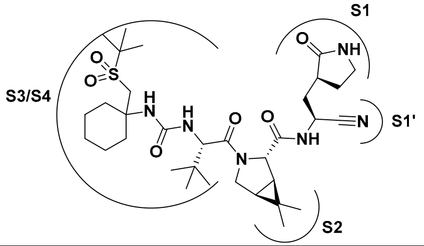	30[d]	Covalent<br>Reversible	1.82	Lead<br>optimization	[5]
14	Alkynes	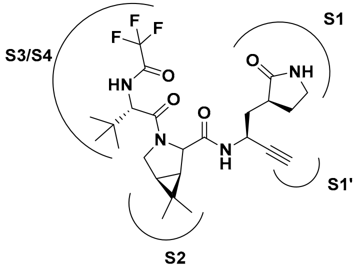	140	Covalent<br>Irreversible	25.7	Lead<br>optimization	[6]
15	Alkynes	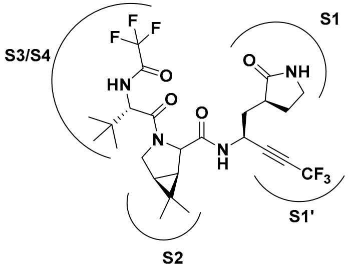	230	Covalent<br>Irreversible	5.1	Lead<br>optimization	[6]

16	Aldehydes	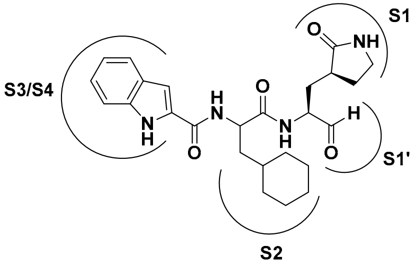	53	Covalent Reversible	0.53	Structure-based design	[7]
17	Aldehydes	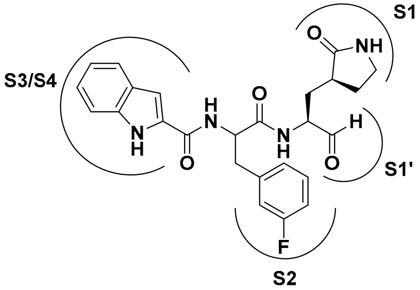	40	Covalent Reversible	0.72	Structure-based design	[7]
18	Aldehydes	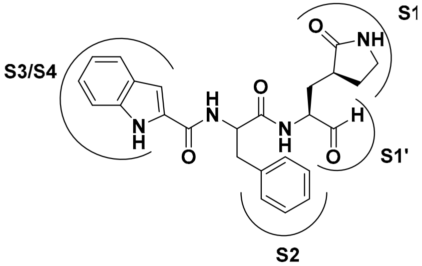	34	Covalent Reversible	0.29	Lead optimization	[8]
19 GC-373	Aldehydes	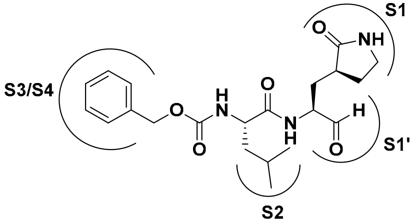	400	Covalent Reversible	1.5	Drug repurposing	[9]

20<br>GC-376	Aldehydes (phosphate prodrug)	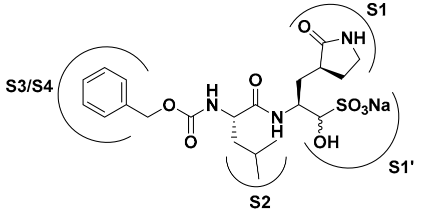	190	Covalent Reversible	0.9	Drug repurposing	[9]
21	Aldehydes (phosphate prodrug)	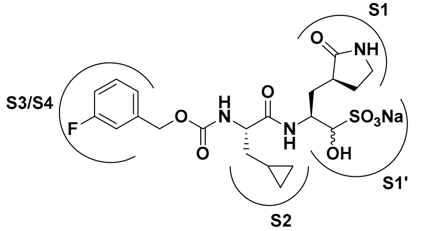	70	Covalent Reversible	0.57	Lead optimization	[10]
22	Aldehydes (phosphate prodrug)	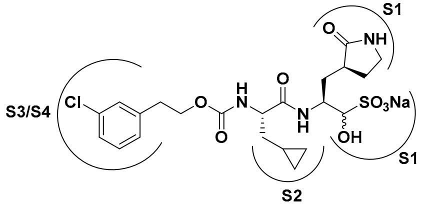	80	Covalent Reversible	0.7	Lead optimization	[10]
23<br>UAWJ247	Aldehydes (phosphate prodrug)	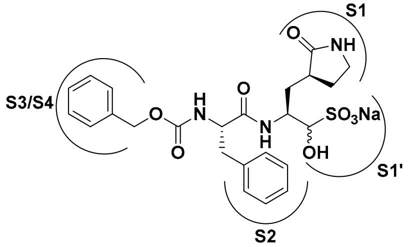	45	Covalent Reversible	6.8	Lead optimization	[11]
24<br>Coronastat	Aldehydes (phosphate prodrug)	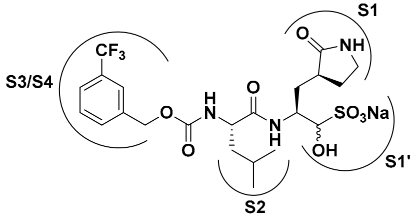	16	Covalent Reversible	0.006	Lead optimization	[12]

25<br>MI-09	Aldehydes	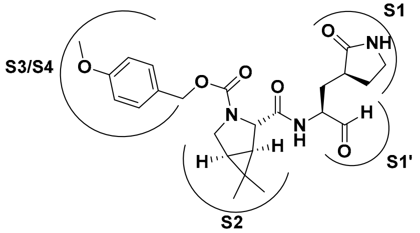	15.2	Covalent<br>Reversible	0.86	Structure-based<br>design	[13]
26<br>Mi-23	Aldehydes	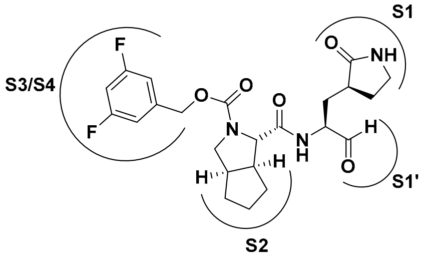	7.6	Covalent<br>Reversible	n.d.	Structure-based<br>design	[13]
27<br>MI-30	Aldehydes	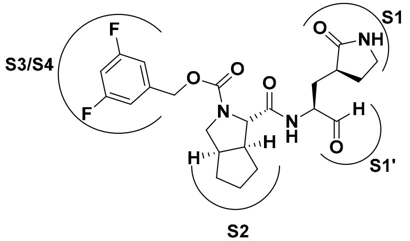	17.2	Covalent<br>Reversible	0.54	Structure-based<br>design	[13]
28	Aldehydes	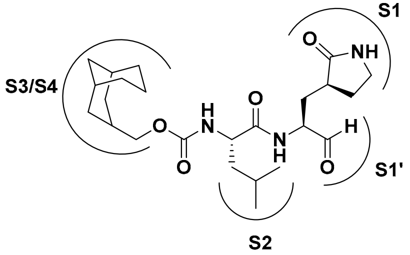	180	Covalent<br>Reversible	0.035	Lead<br>optimization	[14]
29<br>UAWJ9-36-1	Aldehydes	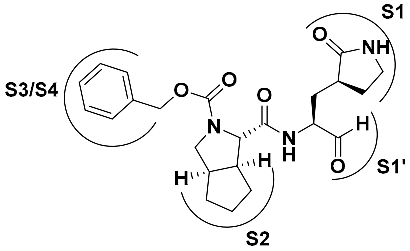	51	Covalent<br>Reversible	2.6	Rational design	[15]

30<br>UAWJ9-36-3	Aldehydes	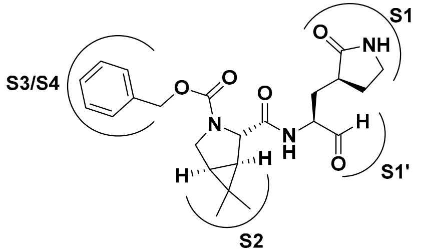	54	Covalent<br>Reversible	0.37	Rational design	[15]
31<br>MPI143	Aldehydes	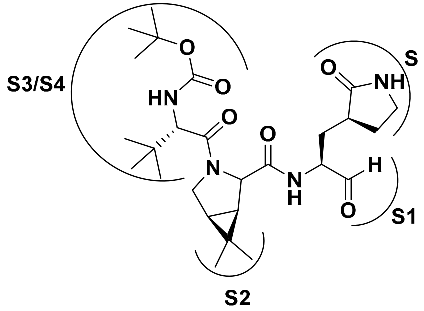	45	Covalent<br>Reversible	0.4 – 1.0[e]	Structure-based design	[16]
32<br>MPI144	Aldehydes	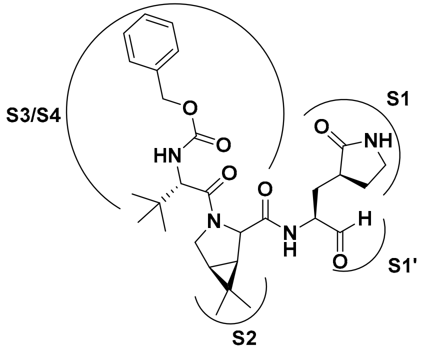	59	Covalent<br>Reversible	0.9 – 2.9[e]	Structure-based design	[16]
33<br>MPI146	Aldehydes	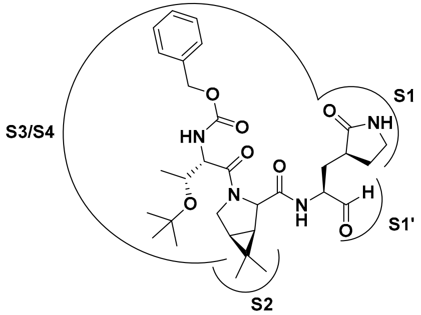	120	Covalent<br>Reversible	0.8 – 2.3[e]	Structure-based design	[16]

34<br>Calpeptin	Aldehydes	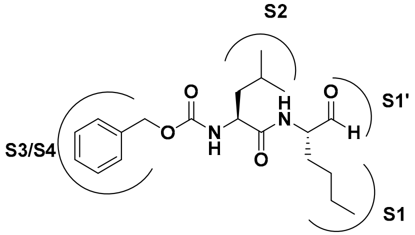	10700	Covalent<br>Reversible	0.072	X-ray screening	[17]
35<br>MPI3	Aldehydes	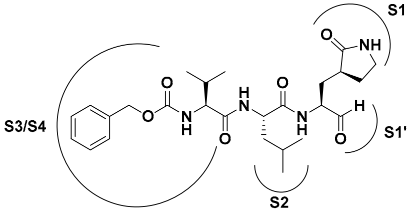	8.5	Covalent<br>Reversible	n.d.	Structure-based<br>design	[18]
36<br>MPI8	Aldehydes	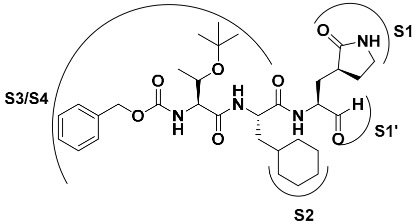	108	Covalent<br>Reversible	2.5	Structure-based<br>design	[18]
37<br>MGI132	Aldehydes	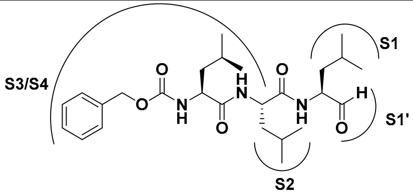	7500	Covalent<br>Reversible	n.d.	Drug<br>repurposing	[19]
38<br>Calpain inhibitor<br>I	Aldehydes	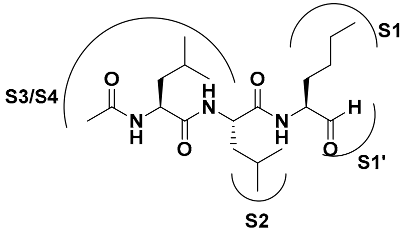	970	Covalent<br>Reversible	n.d.	Drug<br>repurposing	[11]

**39**<br>**Calpain inhibitor II**	**Aldehydes**	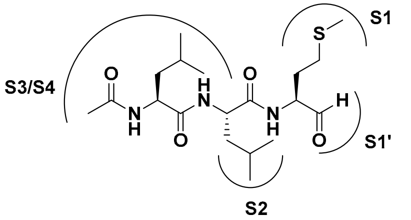	8600	**Covalent Reversible**	**2.07**	**Drug repurposing**	[11]
**40**<br>**MPI16**	**Aldehydes**	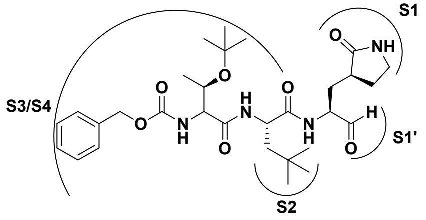	**105**	**Covalent Reversible**	**0.056**	**Lead optimization**	[20]
**41**<br>**MPI17**	**Aldehydes**	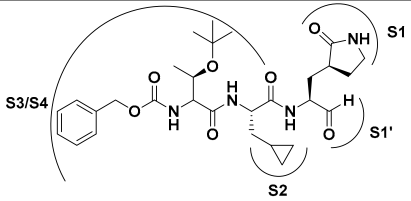	**60**	**Covalent Reversible**	**0.097**	**Lead optimization**	[20]
**42**	**Ketones**	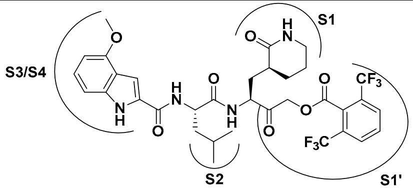	**1.0**	**Covalent Reversible**	**0.16**	**Structure-based design**	[21]
**43**	**Ketones**	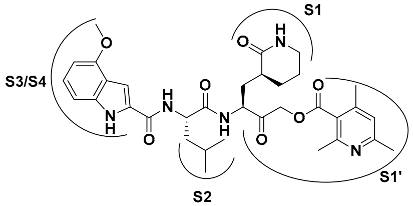	**19.0**	**Covalent Reversible**	**0.3**	**Structure-based design**	[21]
**44**	**Ketones**	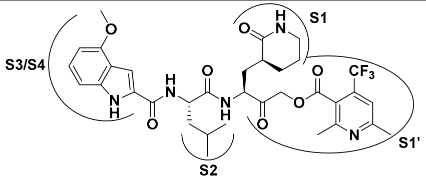	**14.0**	**Covalent Reversible**	**0.47**	**Structure-based design**	[21]

45<br>YH-53	Ketones	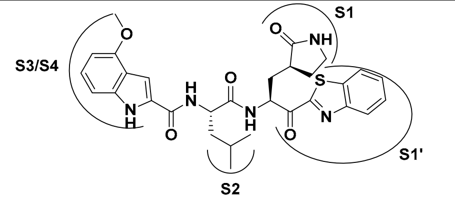	130	Covalent<br>Reversible	2.6	Drug<br>repurposing	[22]
46<br>TKB245	Ketones	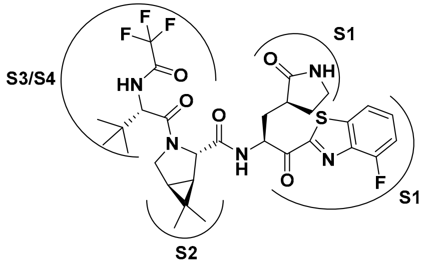	7	Covalent<br>Reversible	0.03	Lead<br>optimization	[23]
47<br>TKB248	Ketones	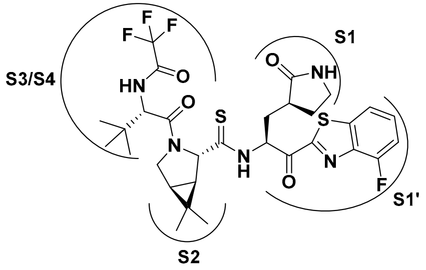	74	Covalent<br>Reversible	0.22	Lead<br>optimization	[23]
48	Ketones	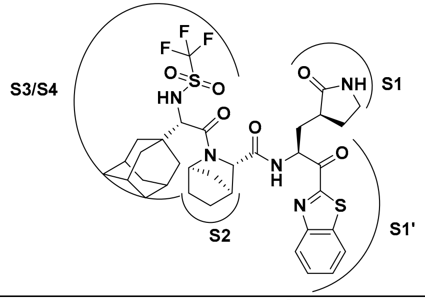	1650	Covalent<br>Reversible	0.18	Lead<br>optimization	[24]
49<br>BBH1	Ketones	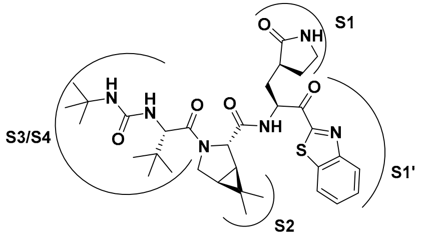	n.d.	Covalent<br>Reversible	16.1	Lead<br>optimization	[5]

**50**<br>**Z-FA-FMK**	**Ketones**	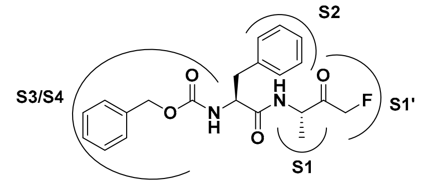	**11400**	**Covalent Irreversible**	**0.13**	**High throughput screening**	[25]
**52**	**Ketones**	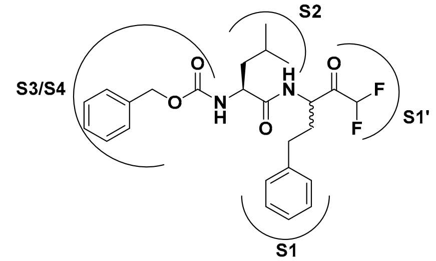	**n.d.**	**Covalent Reversible**	**12.9**[f]	**Warhead modification**	[26]
**53**	**Ketones**	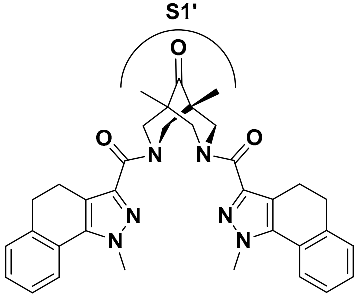	**750**	**Covalent Reversible**	**n.d.**	**Structure-based design**	[27]
**54**<br>**X77**	**Miscellaneous**	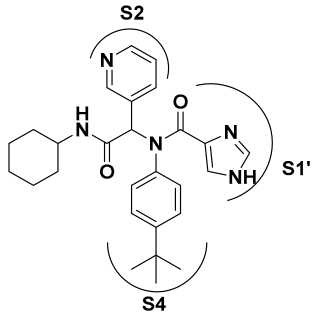	**4100**	**Non Covalent**	**n.d.**	**n.d.**	[28]

55	α-<br>haloacetamides	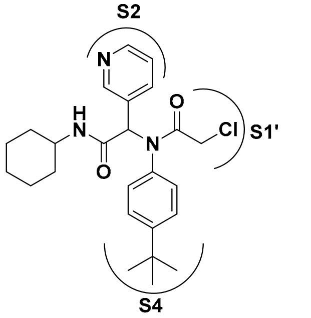	410	Covalent<br>Irreversible	n.d.	Covalent<br>docking	[28]
56<br>23R	Miscellaneous	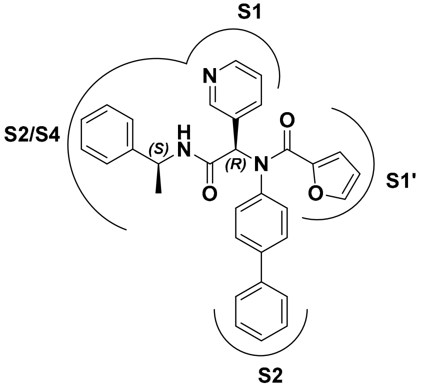	200	Non<br>Covalent	1.4	Structure-based<br>design	[29]
57<br>JUN9-62-2R	α-<br>haloacetamides	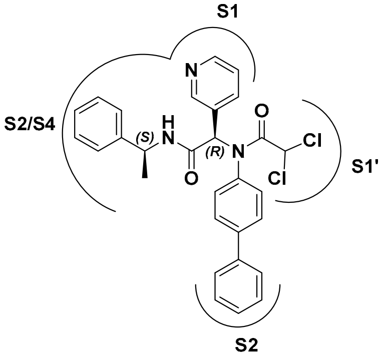	430	Covalent<br>Irreversible	0.90	Warhead<br>replacemnt	[30]

58<br>JUN9-90-3R	α-<br>haloacetamides	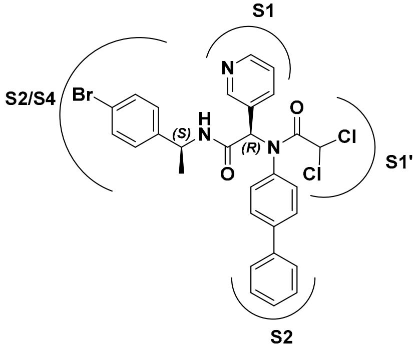	300	Covalent<br>Irreversible	2.07	Warhead<br>replacemnt	[30]
59<br>JUN9-90-4R	α-<br>haloacetamides	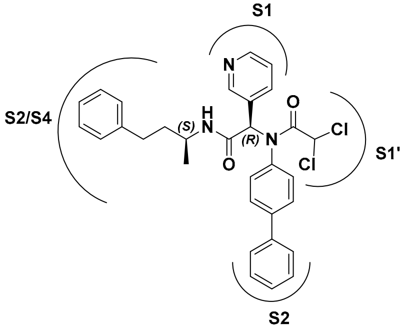	460	Covalent<br>Irreversible	1.10	Warhead<br>replacemnt	[30]
60<br>JUN9-88-6R	α-<br>haloacetamides	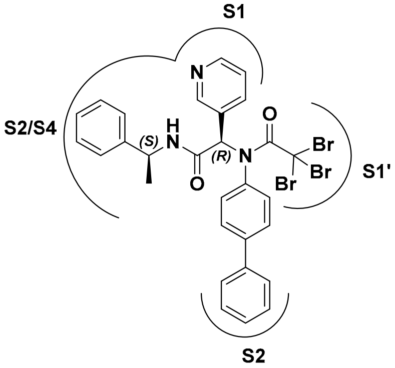	80	Covalent<br>Irreversible	0.58	Warhead<br>replacemnt	[30]

61	α-haloacetamides	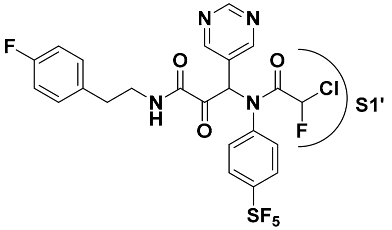	56	Covalent Reversible	n.d.	Lead optimization	[31]
62 Y020-9948	α-haloacetamides	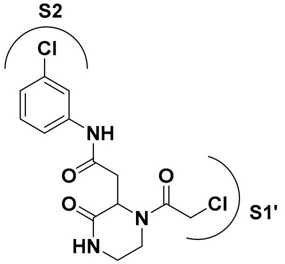	8500	Covalent Irreversible	n.d.	In silico screening and covalent docking	[32]
63 MCULE-5948770040	Miscellaneous	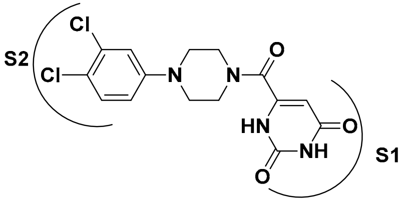	4200	Non Covalent	n.d.	High throughput screening	[33]
64 GC-14	Miscellaneous	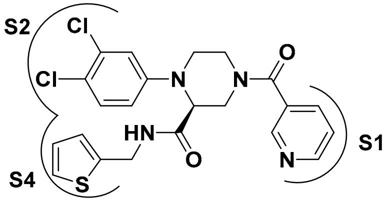	400	Non Covalent	1.1	Structure-based design	[34]
65 GD9	α-haloacetamides	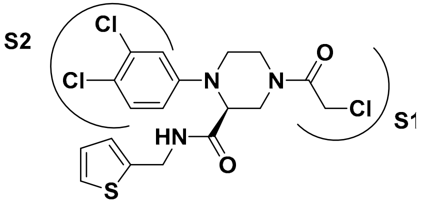	180	Covalent Irreversible	2.6	Warhead replacement	[35]

66<br>GD13	α-<br>haloacetamides	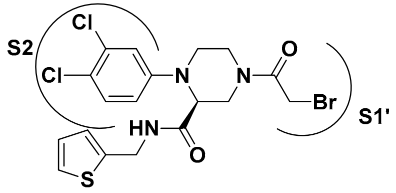	310	Covalent<br>Irreversible	4.2	Warhead<br>replacement	[35]
67<br>(R)-EN82	α-<br>haloacetamides	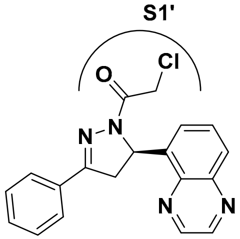	530	Covalent<br>Irreversible	n.d.	Covalent<br>chemoproteomi<br>c strategies and<br>structure-based<br>design	[36]
68<br>HW-2-010b	α-<br>haloacetamides	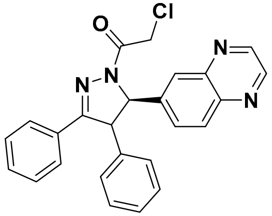	14	Covalent<br>Irreversible	n.d.	Covalent<br>chemoproteomi<br>c strategies and<br>structure-based<br>design	[36]
69<br>QUB-00006-Int-07	α-<br>haloacetamides	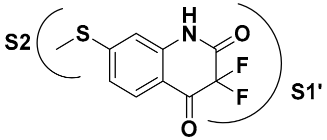	830	Covalent	n.d.	*In silico*<br>strategies	[37]
70	α-ketoamides	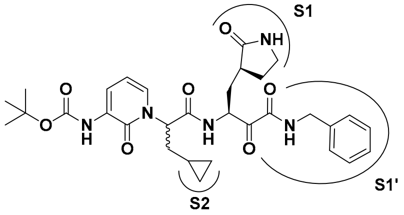	670	Covalent<br>Reversible	4-5	Lead<br>optimization	[38]

**71**<br>**Y180**	**α-ketoamides**	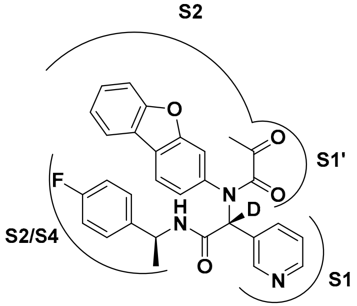	8.1	**Covalent**<br>**Reversible**	**0.011**	**Structure-based**<br>**design**	[39]
**72**<br>**RAY1216**	**α-ketoamides**	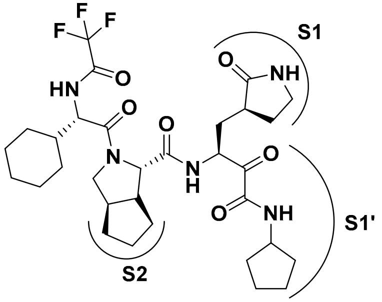	8.4[b]	**Covalent**<br>**Reversible**	**0.13**	**Lead**<br>**optimization**	[40]
**73**	**α-ketoamides**	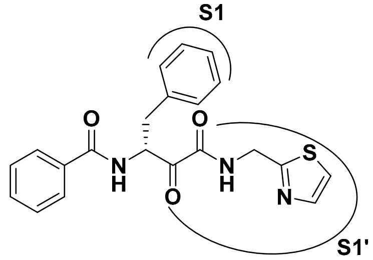	**1300**	**Covalent**<br>**Reversible**	**n.d.**	***In vitro***<br>**screening**	[41]
**74**<br>**SY110**	**α-ketoamides**	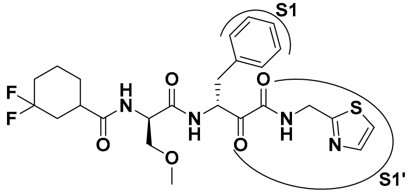	**14.4**	**Covalent**<br>**Reversible**	**0.38 – 2.8[e]**	**Lead**<br>**optimization**	[41]
**75**<br>**N3**	**Michael**<br>**Acceptors**	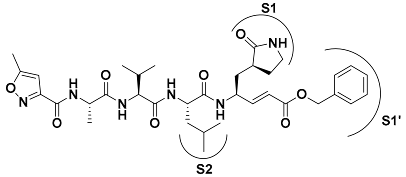	**n.d.**	**Covalent**<br>**Irreversible**	**16.8**	**Computer-**<br>**aided drug**<br>**design**	[42]

**76**<br>**CINANSERIN**	**Michael Acceptors**	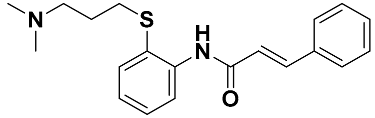	**125000**	**Covalent Irreversible**	**20.6**	**Virtual screening**	[42]
**77**	**Michael Acceptors**	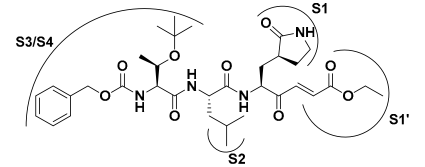	**151**	**Covalent Irreversible**	**2.9**	***In vitro* screening**	[43]
**78**	**Michael Acceptors**	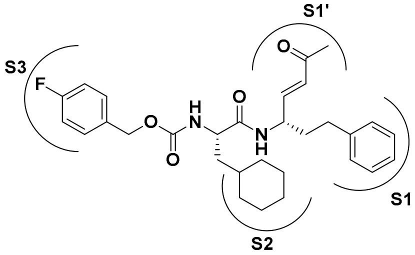	**47200**	**Covalent Irreversible**	**n.d.**	**Virtual screening**	[44]
**79**	**Michael Acceptors**	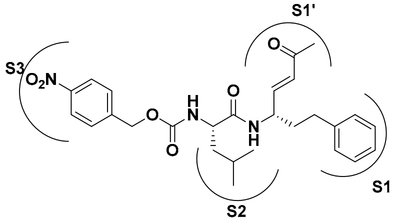	**157000**	**Covalent Irreversible**	**n.d.**	**Virtual screening**	[44]
**80**<br>**SPR38**	**Michael Acceptors**	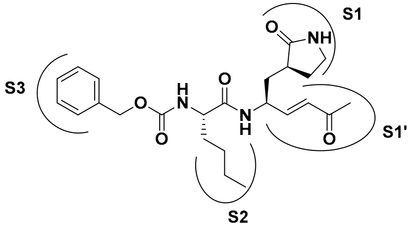	**260[b]**	**Covalent Irreversible**	**18.5**	**Lead optimization**	[45]
**81**<br>**SPR39**	**Michael Acceptors**	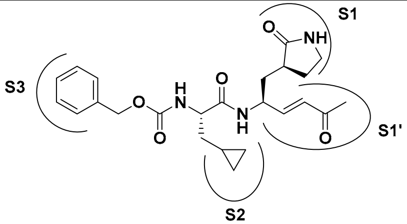	**250[b]**	**Covalent Irreversible**	**1.5**	**Lead optimization**	[45]

82<br>SPR41	Michael<br>Acceptors	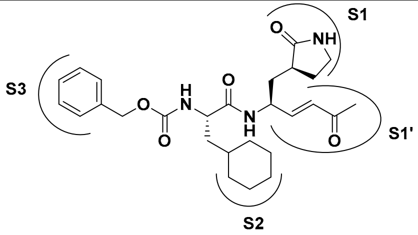	180[b]	Covalent<br>Irreversible	1.8	Lead<br>optimization	[45]
83<br>SM141	Michael<br>Acceptors	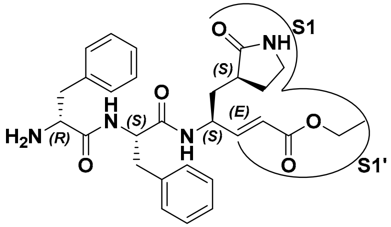	900	Covalent<br>Irreversible	0.0082	Lead<br>optimization	[46]
84<br>SM142	Michael<br>Acceptors	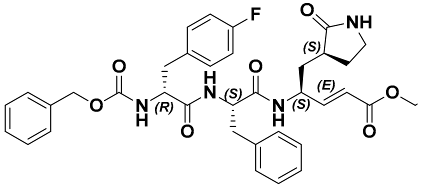	1800	Covalent<br>Irreversible	0.0147	Lead<br>optimization	[46]
85	Michael<br>Acceptors	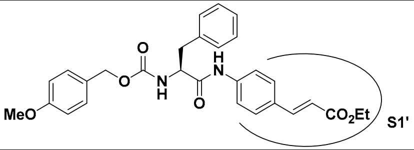	1900	Covalent<br>Irreversible	n.d.	Warhead<br>replacement	[47]
86	Michael<br>Acceptors	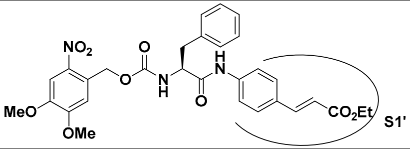	14000	Covalent<br>Irreversible	5.3[f]	Warhead<br>replacement	[47]
87	Michael<br>Acceptors	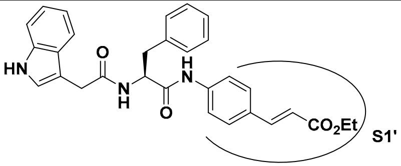	12400	Covalent<br>Irreversible	9.1[g]	Warhead<br>replacement	[47]

88	Michael Acceptors	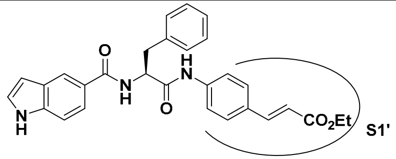	10100	Covalent Irreversible	10.1[g]	Warhead replacement	[47]
89	Michael Acceptors	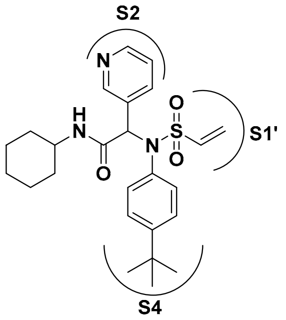	420	Covalent Irreversible	n.d.	Covalent docking	[28]
90	Michael Acceptors	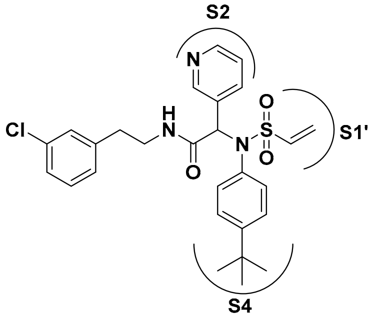	170	Covalent Irreversible	n.d.	Covalent docking	[28]
92	Michael Acceptors	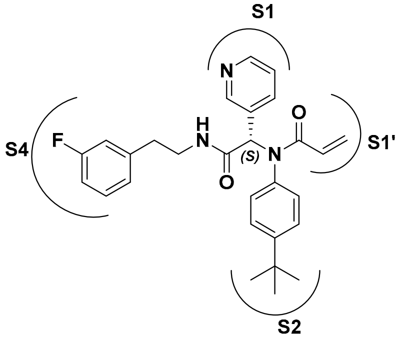	2860	Covalent Irreversible	n.d.	Automatic pipeline	[48]

93<br>MYRECITIN	Michael Acceptors	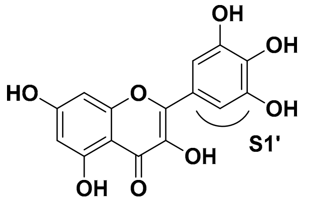	200 - 600	Covalent Irreversible	8	in Vitro Repurposing Screen	[49]
94	Michael Acceptors	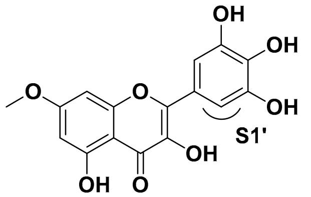	300	Covalent Irreversible	12.6	Structure-based optimization	[50]
95	Michael Acceptors	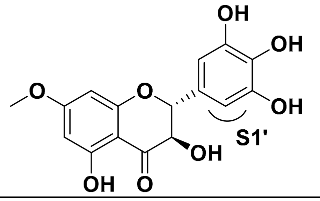	260	Covalent Irreversible	11.5	Structure-based optimization	[50]
96	Michael Acceptors	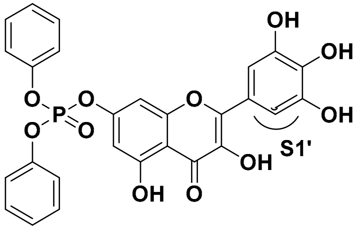	3100	Covalent Irreversible	3.2	Structure-based optimization	[50]
97<br>GRL-0920	Esters	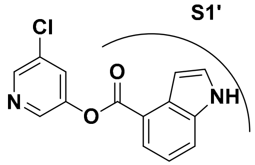	250	Covalent Irreversible	2.8	Drug repurposing	[51]
98<br>GRL-0820	Esters	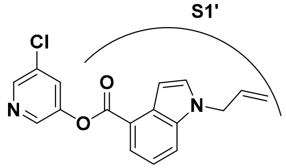	73	Covalent Irreversible	15	Structure-based design	[51]

99<br>GRL-1720	Esters	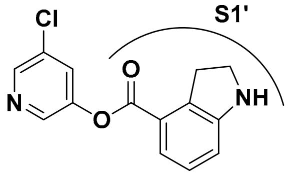	320	Covalent<br>Irreversible	15	Lead<br>optimization	[52]
100	Esters	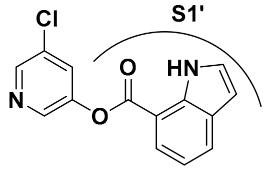	55	Covalent<br>Irreversible	n.d.	Lead<br>optimization	[53]
101	Esters	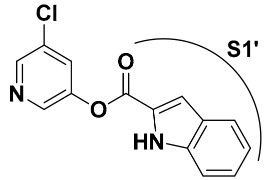	34	Covalent<br>Irreversible	n.d.	Lead<br>optimization	[53]
102	Esters	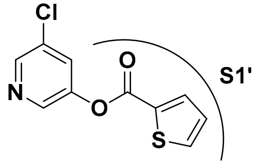	81	Covalent<br>Irreversible	n.d.	*In vitro*<br>screening	[43]
103	Thioesters	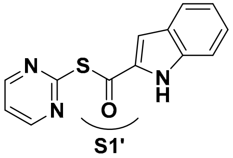	11	Covalent<br>Irreversible	0.11	Virtual<br>screening<br>Structure-based<br>design	[54]
104	Thioesters	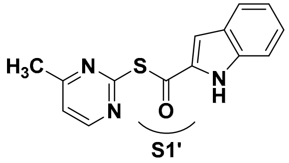	88	Covalent<br>Irreversible	0.73	Virtual<br>screening<br>Structure-based<br>design	[54]
105	Thioesters	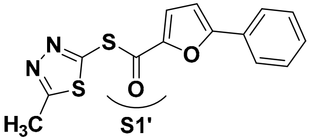	n.d.	n.d.	0.1	Virtual<br>screening<br>Structure-based<br>design	[54]

106	Thioesters	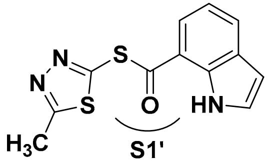	n.d.	n.d.	0.44	Virtual screening Structure-based design	[54]
107	Thioesters	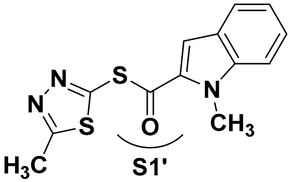	n.d.	n.d.	0.66	Virtual screening Structure-based design	[54]
108	Thioesters	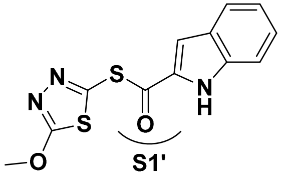	n.d.	n.d.	0.038	Virtual screening Structure-based design	[54]
109	Thioesters	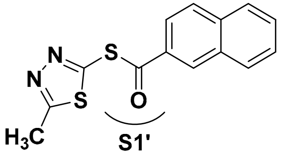	n.d.	n.d.	0.045	Virtual screening Structure-based design	[54]
110 EBSELEN	Selenium-based compounds	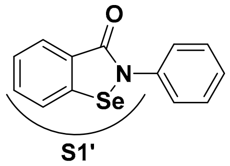	670	Covalent	4.67	Drug repurposing	[42]
111	Selenium-based compounds	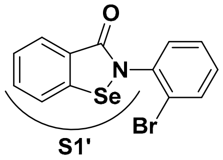	25.7	Covalent	n.d.	*In vitro* screening	[55]
112	Selenium-based compounds	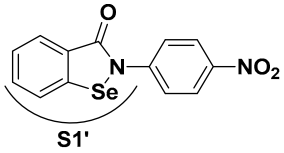	27.9	Covalent	n.d.	*In vitro* screening	[55]

113	Selenium-based compounds	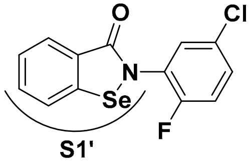	15.2	Covalent	n.d.	*In vitro* screening	[55]
114	Selenium-based compounds	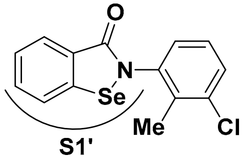	27.4	Covalent	n.d.	*In vitro* screening	[55]
115	Selenium-based compounds	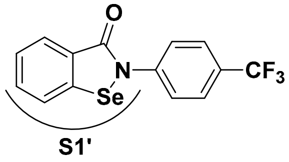	900	Covalent	11.2	Structure-based design	[56]
116	Selenium-based compounds	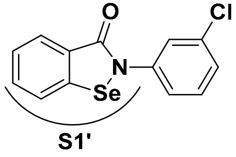	680	Covalent	11.7	Structure-based design	[56]
117	Selenium-based compounds	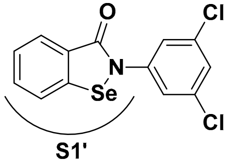	640	Covalent	18.2	Structure-based design	[56]
118	Selenium-based compounds	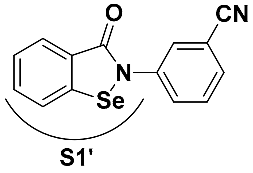	380	Covalent	2.0	Structure-based design	[56]
119	Selenium-based compounds	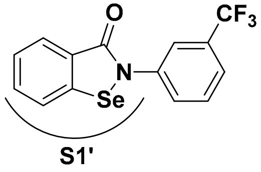	2800	Covalent	0.8	Structure-based design	[56]

120<br>MR6-7-2	Selenium-based compounds	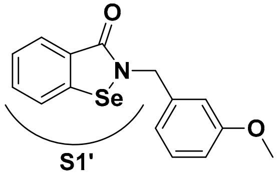 	360	Covalent	4.5	Drug repurposing	[57]
121<br>MR6-18-4	Selenium-based compounds	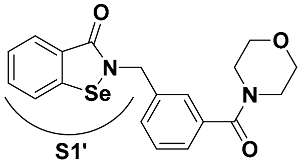 	340	Covalent	3.7	Drug repurposing	[57]
122<br>MR6-17-1	Selenium-based compounds	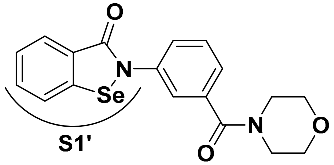 	700	Covalent	n.a.	Drug repurposing	[57]
123<br>MR6-26-2	Selenium-based compounds	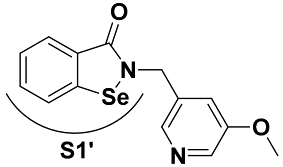 	470	Covalent	3.2	Drug repurposing	[57]
124<br>MR6-31-2	Selenium-based compounds	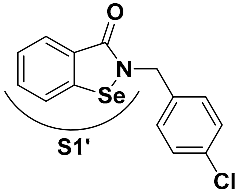 	820	Covalent	1.8	Drug repurposing	[57]
125<br>EBSULFUR	Sulfur-based compounds	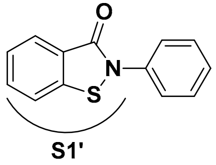 	490	Covalent	n.d.	*In vitro* screening	[58]
126	Sulfur-based compounds	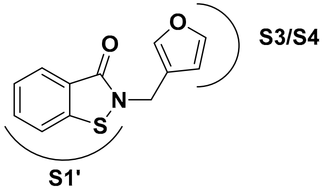 	110	Covalent	n.d.	*In vitro* screening	[58]

**127**	**Selenium-based compounds**	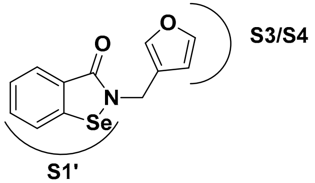	**74**	**Covalent**	**n.d.**	***In vitro* screening**	[58]
**128**	**Sulfur-based compounds**	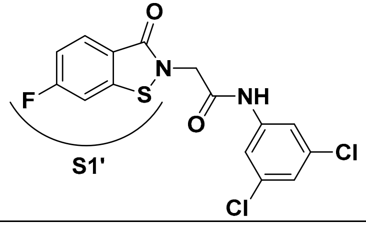	**190**	**Covalent**	**n.d.**	**high-throughput screening**	[59]
**129**	**Sulfur-based compounds**	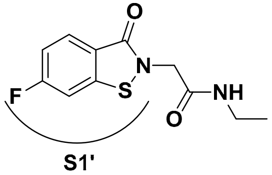	**160**	**Covalent**	**n.d.**	**Lead optimization**	[59]
**130**	**Sulfur-based compounds**	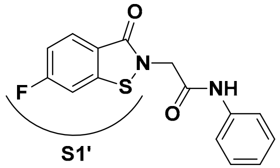	**180**	**Covalent**	**n.d.**	**Lead optimization**	[59]
**131**	**Sulfur-based compounds**	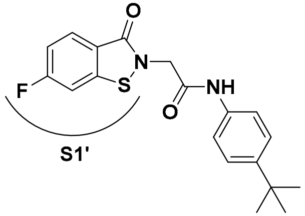	**140**	**Covalent**	**n.d.**	**Lead optimization**	[59]
**132**	**Sulfur-based compounds**	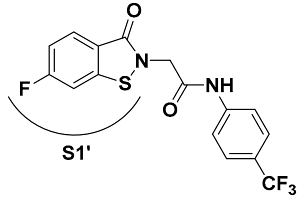	**150**	**Covalent**	**n.d.**	**Lead optimization**	[59]

133	Sulfur-based compounds	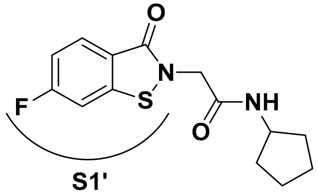	150	Covalent	n.d.	Lead optimization	[59]
134	Sulfur-based compounds	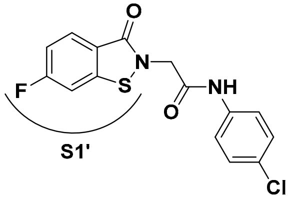	120	Covalent	n.d.	Lead optimization	[59]
135	Sulfur-based compounds	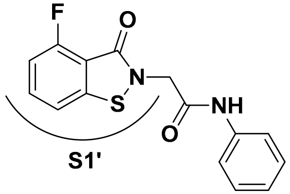	116	Covalent	n.d.	Lead optimization	[59]
136	Sulfur-based compounds	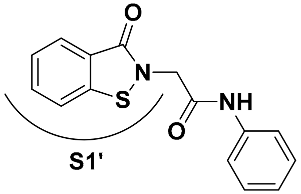	165	Covalent	n.d.	Lead optimization	[59]
137	Sulfur-based compounds	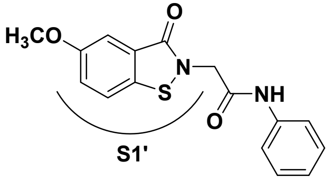	165	Covalent	n.d.	Lead optimization	[59]

138	Miscellaneous	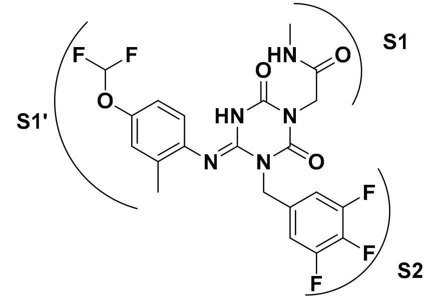	8600	Non Covalent	n.d.	Virtual and *in vitro* screening	[60]
139	Miscellaneous	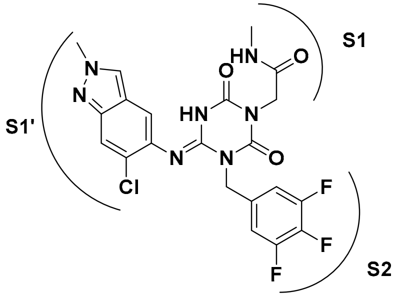	96	Non Covalent	12.5	Lead optimization	[60]
140<br>S-217622	Miscellaneous	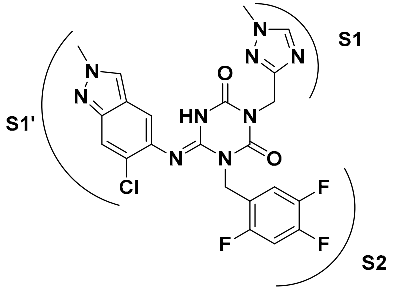	13	Non Covalent	0.5 – 0.29[e]	Lead optimization	[60]
141<br>Z1244904919	Miscellaneous	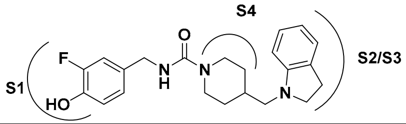	730	Non Covalent	5	Virtual and *in vitro* screening	[61]
142<br>Z1759961356	Miscellaneous	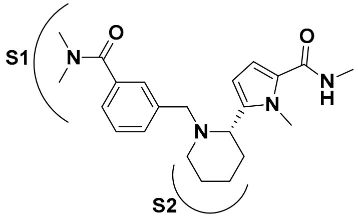	690	Non Covalent	8.5	Virtual and *in vitro* screening	[61]

144<br>CCF981	Miscellaneous	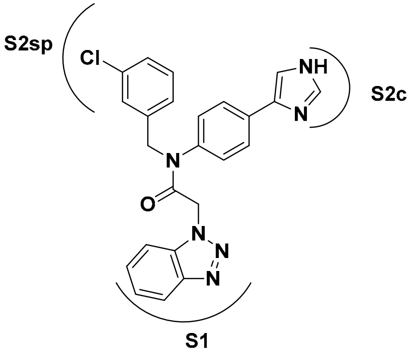	68	Non Covalent	0.5	Lead optimization	[62]
145	Miscellaneous	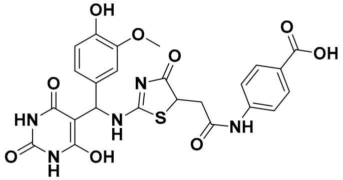	450	Non Covalent	0.77	Virtual and *in vitro* screening	[63]
146	Miscellaneous	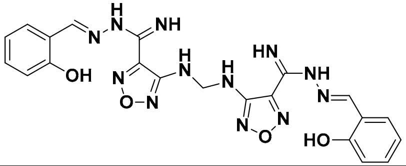	110	Non Covalent	0.11	Virtual and *in vitro* screening	[63]
149	Miscellaneous	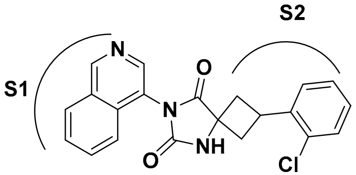	77	Non Covalent	0.11	Lead optimization	[64]
150<br>ZINC00037365906<br>0	Miscellaneous	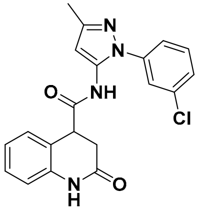	58000	Non Covalent	n.d.	Virtual screening	[65]

151<br>ZINC00063641650<br>1	Miscellaneous	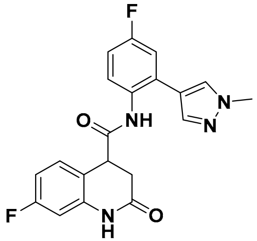	93000	Non<br>Covalent	n.d.	Virtual<br>screening	[65]
152<br>Z228770960	Miscellaneous	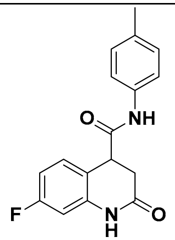	4000	Non<br>Covalent	n.d.	Virtual<br>screening	[65]
153<br>Z393665558	Miscellaneous	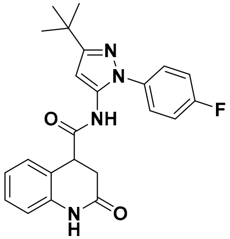	6000	Non<br>Covalent	n.d.	Virtual<br>screening	[65]
154<br>Z225602086	Miscellaneous	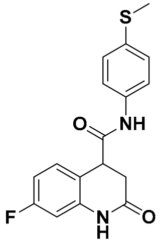	7400	Non<br>Covalent	n.d.	Virtual<br>screening	[65]
155<br>Z222979552	Miscellaneous	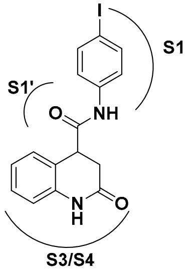	1000	Non<br>Covalent	n.d.	Virtual<br>screening	[65]

156<br>Z228166018	Miscellaneous	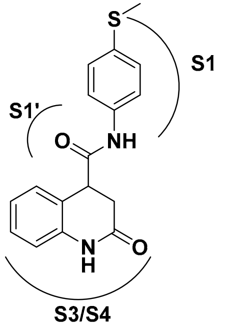	1600	Non Covalent	n.d.	Virtual screening	[65]
157<br>Z222977344	Miscellaneous	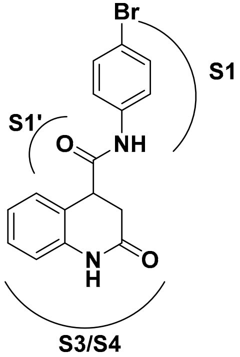	2000	Non Covalent	n.d.	Virtual screening	[65]
158<br>Z222978028	Miscellaneous	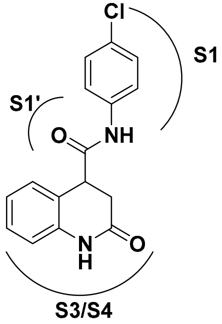	5800	Non Covalent	n.d.	Virtual screening	[65]
160	Miscellaneous	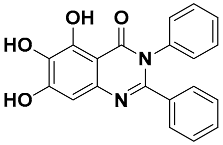	83	Non Covalent	1.1	Structure-based design	[66]

161	Miscellaneous	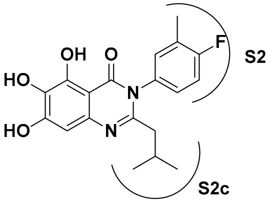	100	Non Covalent	2.1	Structure-based design	[66]
162	Miscellaneous	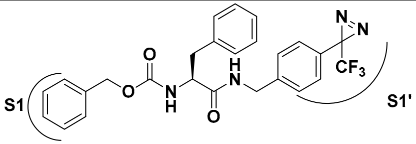	4100	Non Covalent	n.d.	Warhead replacement	[67]
163 Neoechinulin A	Miscellaneous	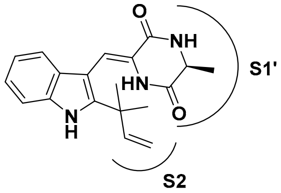	470	Non Covalent	n.d.	*in vitro* screening	[68]
164 Shikonin	Miscellaneous	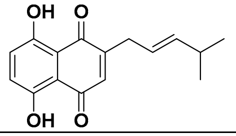	1570	Non Covalent	n.d.	Virtual screening	[69]
165 C1	Miscellaneous	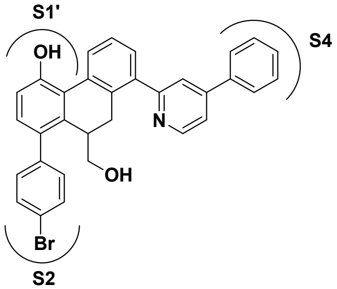	1500	Non Covalent	n.d.	Lead optimization	[70]
166 C2	Miscellaneous	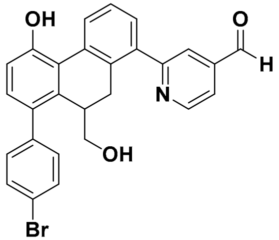	1800	Non Covalent	n.d.	Lead optimization	[70]

**167**<br>**Pelitinib**	**Miscellaneous**	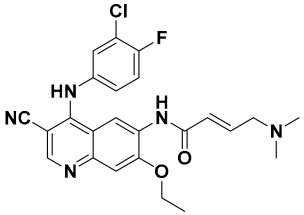	**n.d.**	**Allosteric**	**1.25**	**X-ray screening**<br>**Drug**<br>**repurposing**	[17]
**168**<br>**Ifenprodil**	**Miscellaneous**	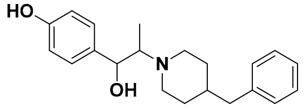	**n.d.**	**Allosteric**	**47**	**X-ray screening**<br>**Drug**<br>**repurposing**	[17]
**169**<br>**RS-102895**	**Miscellaneous**	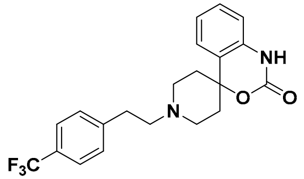	**n.d.**	**Allosteric**	**19.8**	**X-ray screening**<br>**Drug**<br>**repurposing**	[17]
**170**<br>**AT7519**	**Miscellaneous**	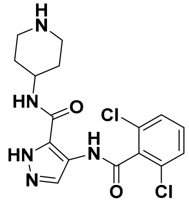	**n.d.**	**Allosteric**	**25.2**	**X-ray screening**<br>**Drug**<br>**repurposing**	[17]
**171**<br>**JMX0286**	**Miscellaneous**	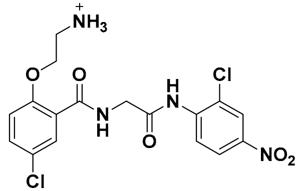	**4800**	**Allosteric**	**2.3**	***In vitro***<br>**screening**	[71]
**172**<br>**JMX0301**	**Miscellaneous**	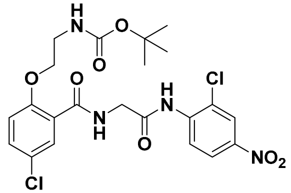	**4500**	**Allosteric**	**2.4**	***In vitro***<br>**screening**	[71]
**173**<br>**JMX0941**	**Miscellaneous**	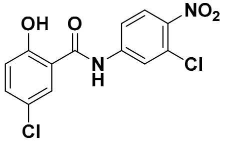	**3900**	**Allosteric**	**1.7**	***In vitro***<br>**screening**	[71]

**174**<br>**Apixaban**	**Miscellaneous**	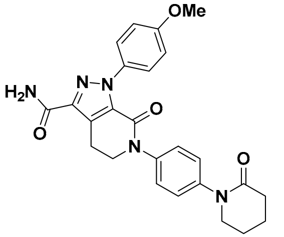	**9.7**	**Allosteric**	**1.8**	**Drug repurposing**	[72]

References

[1] D. R. Owen *et al.*, 'An oral SARS-CoV-2 M pro inhibitor clinical candidate for the treatment of COVID-19', *Science (1979)*, vol. 374, no. 6575, pp. 1586–1593, Dec. 2021, doi: 10.1126/science.abl4784.

[2] 'WO2021250648A1 - Nitrile-containing antiviral compounds - Google Patents'. https://patents.google.com/patent/WO2021250648A1/en (accessed Mar. 05, 2023).

[3] B. Bai *et al.*, 'Peptidomimetic nitrile warheads as SARS-CoV-2 3CL protease inhibitors', *RSC Med Chem*, vol. 12, no. 10, pp. 1722–1730, 2021, doi: 10.1039/D1MD00247C.

[4] M. Zhu *et al.*, 'Design, synthesis and biological evaluation of covalent peptidomimetic 3CL protease inhibitors containing nitrile moiety', *Bioorg Med Chem*, vol. 87, p. 117316, May 2023, doi: 10.1016/J.BMC.2023.117316.

[5] D. W. Kneller *et al.*, 'Covalent narlaprevir- and boceprevir-derived hybrid inhibitors of SARS-CoV-2 main protease', *Nat Commun*, vol. 13, no. 1, p. 2268, Apr. 2022, doi: 10.1038/s41467-022-29915-z.

[6] L. Brewitz *et al.*, 'Alkyne Derivatives of SARS-CoV-2 Main Protease Inhibitors Including Nirmatrelvir Inhibit by Reacting Covalently with the Nucleophilic Cysteine', *J Med Chem*, vol. 66, no. 4, pp. 2663–2680, Feb. 2023, doi: 10.1021/acs.jmedchem.2c01627.

[7] W. Dai *et al.*, 'Structure-based design of antiviral drug candidates targeting the SARS-CoV-2 main protease', *Science (1979)*, vol. 368, no. 6497, pp. 1331–1335, Jun. 2020, doi: 10.1126/science.abb4489.

[8] W. Dai *et al.*, 'Design, Synthesis, and Biological Evaluation of Peptidomimetic Aldehydes as Broad-Spectrum Inhibitors against Enterovirus and SARS-CoV-2', *J Med Chem*, vol. 65, no. 4, pp. 2794–2808, Feb. 2022, doi: 10.1021/acs.jmedchem.0c02258.

[9] W. Vuong *et al.*, 'Feline coronavirus drug inhibits the main protease of SARS-CoV-2 and blocks virus replication', *Nat Commun*, vol. 11, no. 1, p. 4282, Aug. 2020, doi: 10.1038/s41467-020-18096-2.

[10] W. Vuong *et al.*, 'Improved SARS-CoV-2 Mpro inhibitors based on feline antiviral drug GC376: Structural enhancements, increased solubility, and micellar studies', *Eur J Med Chem*, vol. 222, p. 113584, Oct. 2021, doi: 10.1016/j.ejmech.2021.113584.

[11] M. D. Sacco *et al.*, 'Structure and inhibition of the SARS-CoV-2 main protease reveal strategy for developing dual inhibitors against Mpro and cathepsin L', *Sci Adv*, vol. 6, no. 50, Dec. 2020, doi: 10.1126/SCIADV.ABE0751/SUPPL_FILE/ABE0751_SM.PDF.

[12] H. Liu *et al.*, 'Development of optimized drug-like small molecule inhibitors of the SARS-CoV-2 3CL protease for treatment of COVID-19', *Nat Commun*, vol. 13, no. 1, p. 1891, Apr. 2022, doi: 10.1038/s41467-022-29413-2.

[13] J. Qiao *et al.*, 'SARS-CoV-2 M pro inhibitors with antiviral activity in a transgenic mouse model', *Science (1979)*, vol. 371, no. 6536, pp. 1374–1378, Mar. 2021, doi: 10.1126/science.abf1611.

[14] C. S. Dampalla *et al.*, 'Structure-Guided Design of Conformationally Constrained Cyclohexane Inhibitors of Severe Acute Respiratory Syndrome Coronavirus-2 3CL Protease', *J Med Chem*, vol. 64, no. 14, pp. 10047–10058, Jul. 2021, doi: 10.1021/acs.jmedchem.1c00319.

[15] Z. Xia *et al.*, 'Rational Design of Hybrid SARS-CoV-2 Main Protease Inhibitors Guided by the Superimposed Cocrystal Structures with the Peptidomimetic Inhibitors GC-376, Telaprevir, and Boceprevir', *ACS Pharmacol Transl Sci*, vol. 4, no. 4, pp. 1408–1421, Aug. 2021, doi: 10.1021/acsptsci.1c00099.

[16] Y. R. Alugubelli *et al.*, 'A systematic exploration of boceprevir-based main protease inhibitors as SARS-CoV-2 antivirals', *Eur J Med Chem*, vol. 240, p. 114596, Oct. 2022, doi: 10.1016/j.ejmech.2022.114596.

[17] S. Günther *et al.*, 'X-ray screening identifies active site and allosteric inhibitors of SARS-CoV-2 main protease', *Science (1979)*, vol. 372, no. 6542, pp. 642–646, May 2021, doi: 10.1126/science.abf7945.

[18] K. S. Yang *et al.*, 'A Quick Route to Multiple Highly Potent SARS-CoV-2 Main Protease Inhibitors**', *ChemMedChem*, vol. 16, no. 6, pp. 942–948, Mar. 2021, doi: 10.1002/cmdc.202000924.

[19] E. Costanzi *et al.*, 'Structural and Biochemical Analysis of the Dual Inhibition of MG-132 against SARS-CoV-2 Main Protease (Mpro/3CLpro) and Human Cathepsin-L', *Int J Mol Sci*, vol. 22, no. 21, p. 11779, Oct. 2021, doi: 10.3390/ijms222111779.

[20] Y. Ma *et al.*, 'A multi-pronged evaluation of aldehyde-based tripeptidyl main protease inhibitors as SARS-CoV-2 antivirals', *Eur J Med Chem*, vol. 240, p. 114570, Oct. 2022, doi: 10.1016/j.ejmech.2022.114570.

[21] B. Bai *et al.*, 'Peptidomimetic α-Acyloxymethylketone Warheads with Six-Membered Lactam P1 Glutamine Mimic: SARS-CoV-2 3CL Protease Inhibition, Coronavirus Antiviral Activity, and *in Vitro* Biological Stability', *J Med Chem*, vol. 65, no. 4, pp. 2905–2925, Feb. 2022, doi: 10.1021/acs.jmedchem.1c00616.

[22] S. Hattori *et al.*, 'A small molecule compound with an indole moiety inhibits the main protease of SARS-CoV-2 and blocks virus replication', *Nat Commun*, vol. 12, no. 1, p. 668, Jan. 2021, doi: 10.1038/s41467-021-20900-6.

[23] N. Higashi-Kuwata *et al.*, 'Identification of SARS-CoV-2 Mpro inhibitors containing P1' 4-fluorobenzothiazole moiety highly active against SARS-CoV-2', *Nat Commun*, vol. 14, no. 1, p. 1076, Feb. 2023, doi: 10.1038/s41467-023-36729-0.

[24] H. Yang *et al.*, 'Design, synthesis and biological evaluation of peptidomimetic benzothiazolyl ketones as 3CLpro inhibitors against SARS-CoV-2', *Eur J Med Chem*, vol. 257, p. 115512, Sep. 2023, doi: 10.1016/J.EJMECH.2023.115512.

[25] W. Zhu *et al.*, 'Identification of SARS-CoV-2 3CL Protease Inhibitors by a Quantitative High-Throughput Screening', *ACS Pharmacol Transl Sci*, vol. 3, no. 5, pp. 1008–1016, Oct. 2020, doi: 10.1021/acsptsci.0c00108.

[26] A. Citarella *et al.*, 'Pseudo-Dipeptide Bearing α,α-Difluoromethyl Ketone Moiety as Electrophilic Warhead with Activity against Coronaviruses', *International Journal of Molecular Sciences 2021, Vol. 22, Page 1398*, vol. 22, no. 3, p. 1398, Jan. 2021, doi: 10.3390/IJMS22031398.

[27] D. Shcherbakov *et al.*, 'Design and Evaluation of Bispidine-Based SARS-CoV-2 Main Protease Inhibitors', *ACS Med Chem Lett*, vol. 13, no. 1, pp. 140–147, Jan. 2022, doi: 10.1021/acsmedchemlett.1c00299.

[28] J. K. Stille *et al.*, 'Design, synthesis and in vitro evaluation of novel SARS-CoV-2 3CLpro covalent inhibitors', *Eur J Med Chem*, vol. 229, p. 114046, Feb. 2022, doi: 10.1016/J.EJMECH.2021.114046.

[29] N. Kitamura *et al.*, 'Expedited Approach toward the Rational Design of Noncovalent SARS-CoV-2 Main Protease Inhibitors', *J Med Chem*, vol. 65, no. 4, pp. 2848–2865, Feb. 2022, doi: 10.1021/ACS.JMEDCHEM.1C00509/SUPPL_FILE/JM1C00509_SI_002.CSV.

[30] C. Ma *et al.*, 'Discovery of Di- and Trihaloacetamides as Covalent SARS-CoV-2 Main Protease Inhibitors with High Target Specificity', *J Am Chem Soc*, vol. 143, no. 49, pp. 20697–20709, Dec. 2021, doi: 10.1021/jacs.1c08060.

[31] D. Yamane *et al.*, 'Selective covalent targeting of SARS-CoV-2 main protease by enantiopure chlorofluoroacetamide', *Chem Sci*, vol. 13, no. 10, pp. 3027–3034, 2022, doi: 10.1039/D1SC06596C.

[32] M. Xiong, T. Nie, Q. Shao, M. Li, H. Su, and Y. Xu, 'In silico screening-based discovery of novel covalent inhibitors of the SARS-CoV-2 3CL protease', *Eur J Med Chem*, vol. 231, p. 114130, Mar. 2022, doi: 10.1016/j.ejmech.2022.114130.

[33] A. Clyde *et al.*, 'High-Throughput Virtual Screening and Validation of a SARS-CoV-2 Main Protease Noncovalent Inhibitor', *J Chem Inf Model*, vol. 62, no. 1, pp. 116–128, Jan. 2022, doi: 10.1021/ACS.JCIM.1C00851/ASSET/IMAGES/LARGE/CI1C00851_0005.JPEG.

[34] S. Gao *et al.*, 'Discovery and Crystallographic Studies of Trisubstituted Piperazine Derivatives as Non-Covalent SARS-CoV-2 Main Protease Inhibitors with High Target Specificity and Low Toxicity', *J Med Chem*, vol. 65, no. 19, pp. 13343–13364, Oct. 2022, doi: 10.1021/ACS.JMEDCHEM.2C01146/SUPPL_FILE/JM2C01146_SI_002.CSV.

[35] S. Gao *et al.*, 'Discovery and Crystallographic Studies of Nonpeptidic Piperazine Derivatives as Covalent SARS-CoV-2 Main Protease Inhibitors', *J Med Chem*, vol. 65, no. 24, pp. 16902–16917, Dec. 2022, doi: 10.1021/ACS.JMEDCHEM.2C01716/SUPPL_FILE/JM2C01716_SI_001.CSV.

[36] P. Moon *et al.*, 'Discovery of Potent Pyrazoline-Based Covalent SARS-CoV-2 Main Protease Inhibitors', *bioRxiv*, p. 2022.03.05.483025, Mar. 2022, doi: 10.1101/2022.03.05.483025.

[37] L. El Khoury *et al.*, 'Computationally driven discovery of SARS-CoV-2 Mpro inhibitors: from design to experimental validation', *Chem Sci*, vol. 13, no. 13, pp. 3674–3687, Mar. 2022, doi: 10.1039/D1SC05892D.

[38] L. Zhang *et al.*, 'Crystal structure of SARS-CoV-2 main protease provides a basis for design of improved α-ketoamide inhibitors', *Science (1979)*, vol. 368, no. 6489, pp. 409–412, Apr. 2020, doi: 10.1126/science.abb3405.

[39] B. X. Quan *et al.*, 'An orally available Mpro inhibitor is effective against wild-type SARS-CoV-2 and variants including Omicron', *Nature Microbiology 2022 7:5*, vol. 7, no. 5, pp. 716–725, Apr. 2022, doi: 10.1038/s41564-022-01119-7.

[40] X. Chen *et al.*, 'Inhibition mechanism and antiviral activity of an α-ketoamide based SARS-CoV-2 main protease inhibitor', *bioRxiv*, p. 2023.03.09.531862, Mar. 2023, doi: 10.1101/2023.03.09.531862.

[41] C. Huang *et al.*, 'A new generation Mpro inhibitor with potent activity against SARS-CoV-2 Omicron variants', *Signal Transduction and Targeted Therapy 2023 8:1*, vol. 8, no. 1, pp. 1–13, Mar. 2023, doi: 10.1038/s41392-023-01392-w.

[42] Z. Jin *et al.*, 'Structure of Mpro from SARS-CoV-2 and discovery of its inhibitors', *Nature 2020 582:7811*, vol. 582, no. 7811, pp. 289–293, Apr. 2020, doi: 10.1038/s41586-020-2223-y.

[43] S. Iketani *et al.*, 'Lead compounds for the development of SARS-CoV-2 3CL protease inhibitors', *Nature Communications 2021 12:1*, vol. 12, no. 1, pp. 1–7, Apr. 2021, doi: 10.1038/s41467-021-22362-2.

[44] G. Amendola *et al.*, 'Lead Discovery of SARS-CoV-2 Main Protease Inhibitors through Covalent Docking-Based Virtual Screening', *J Chem Inf Model*, vol. 61, no. 4, pp. 2062–2073, Apr. 2021, doi: 10.1021/acs.jcim.1c00184.

[45] S. Previti *et al.*, 'Structure-based lead optimization of peptide-based vinyl methyl ketones as SARS-CoV-2 main protease inhibitors', *Eur J Med Chem*, vol. 247, p. 115021, Feb. 2023, doi: 10.1016/j.ejmech.2022.115021.

[46] S. Mondal *et al.*, 'Dual Inhibitors of Main Protease (M ^{Pro}) and Cathepsin L as Potent Antivirals against SARS-CoV2', *J Am Chem Soc*, vol. 144, no. 46, pp. 21035–21045, Nov. 2022, doi: 10.1021/jacs.2c04626.

[47] A. Citarella *et al.*, 'Synthesis of SARS-CoV-2 Mpro inhibitors bearing a cinnamic ester warhead with in vitro activity against human coronaviruses', *Org Biomol Chem*, 2023, doi: 10.1039/D3OB00381G.

[48] D. Zaidman, P. Gehrtz, M. Filep, M. A. Walsh, F. Von Delft, and N. London Correspondence, 'An automatic pipeline for the design of irreversible derivatives identifies a potent SARS-CoV-2 M pro inhibitor', *Cell Chem Biol*, vol. 28, pp. 1795-1806.e5, 2021, doi: 10.1016/j.chembiol.2021.05.018.

[49] M. Kuzikov *et al.*, 'Identification of Inhibitors of SARS-CoV-2 3CL-Pro Enzymatic Activity Using a Small Molecule in Vitro Repurposing Screen', *ACS Pharmacol Transl Sci*, vol. 4, no. 3, pp. 1096–1110, Jun. 2021, doi: 10.1021/ACSPTSCI.0C00216/ASSET/IMAGES/LARGE/PT0C00216_0007.JPEG.

[50] H. Su *et al.*, 'Identification of pyrogallol as a warhead in design of covalent inhibitors for the SARS-CoV-2 3CL protease', *Nature Communications 2021 12:1*, vol. 12, no. 1, pp. 1–12, Jun. 2021, doi: 10.1038/s41467-021-23751-3.

[51] S. I. Hattori *et al.*, 'GRL-0920, an indole chloropyridinyl ester, completely blocks SARS-CoV-2 infection', *mBio*, vol. 11, no. 4, pp. 1–16, Jul. 2020, doi: 10.1128/MBIO.01833-20/ASSET/11D12B15-E56A-4381-A5F9-AD6A79F97BE2/ASSETS/GRAPHIC/MBIO.01833-20-F0005.JPEG.

[52] A. K. Ghosh *et al.*, 'Indole Chloropyridinyl Ester-Derived SARS-CoV-2 3CLpro Inhibitors: Enzyme Inhibition, Antiviral Efficacy, Structure-Activity Relationship, and X-ray Structural Studies', *J Med Chem*, vol. 64, no. 19, pp. 14702–14714, Oct. 2021, doi: 10.1021/ACS.JMEDCHEM.1C01214/SUPPL_FILE/JM1C01214_SI_002.CSV.

[53] J. Breidenbach *et al.*, 'Targeting the Main Protease of SARS-CoV-2: From the Establishment of High Throughput Screening to the Design of Tailored Inhibitors', *Angewandte Chemie International Edition*, vol. 60, no. 18, pp. 10423–10429, Apr. 2021, doi: 10.1002/ANIE.202016961.

[54] T. Pillaiyar *et al.*, 'Small-Molecule Thioesters as SARS-CoV-2 Main Protease Inhibitors: Enzyme Inhibition, Structure–Activity Relationships, Antiviral Activity, and X-ray Structure Determination', *J Med Chem*, vol. 65, no. 13, pp. 9376–9395, Jul. 2022, doi: 10.1021/acs.jmedchem.2c00636.

[55] M. Zmudzinski *et al.*, 'Ebselen derivatives are very potent dual inhibitors of SARS-CoV-2 proteases - PLpro and Mpro in in vitro studies', *bioRxiv*, p. 2020.08.30.273979, Aug. 2020, doi: 10.1101/2020.08.30.273979.

[56] S. Huff *et al.*, 'Discovery and Mechanism of SARS-CoV-2 Main Protease Inhibitors', *J Med Chem*, vol. 65, no. 4, pp. 2866–2879, Feb. 2022, doi: 10.1021/ACS.JMEDCHEM.1C00566/SUPPL_FILE/JM1C00566_SI_001.CSV.

[57] K. Amporndanai *et al.*, 'Inhibition mechanism of SARS-CoV-2 main protease by ebselen and its derivatives', *Nature Communications 2021 12:1*, vol. 12, no. 1, pp. 1–7, May 2021, doi: 10.1038/s41467-021-23313-7.

[58] L. Y. Sun *et al.*, 'Ebsulfur and Ebselen as highly potent scaffolds for the development of potential SARS-CoV-2 antivirals', *Bioorg Chem*, vol. 112, p. 104889, Jul. 2021, doi: 10.1016/J.BIOORG.2021.104889.

[59] W. Chen *et al.*, 'Discovery of highly potent SARS-CoV-2 Mpro inhibitors based on benzoisothiazolone scaffold', *Bioorg Med Chem Lett*, vol. 58, p. 128526, Feb. 2022, doi: 10.1016/j.bmcl.2022.128526.

[60] Y. Unoh *et al.*, 'Discovery of S-217622, a Noncovalent Oral SARS-CoV-2 3CL Protease Inhibitor Clinical Candidate for Treating COVID-19', *J Med Chem*, vol. 65, no. 9, pp. 6499–6512, May 2022, doi: 10.1021/acs.jmedchem.2c00117.

[61] J. Yang *et al.*, 'Structure-Based Discovery of Novel Nonpeptide Inhibitors Targeting SARS-CoV-2 M ^{pro}', *J Chem Inf Model*, vol. 61, no. 8, pp. 3917–3926, Aug. 2021, doi: 10.1021/acs.jcim.1c00355.

[62] S. H. Han *et al.*, 'Structure-Based Optimization of ML300-Derived, Noncovalent Inhibitors Targeting the Severe Acute Respiratory Syndrome Coronavirus 3CL Protease (SARS-CoV-2 3CLpro)', *J Med Chem*, vol. 65, no. 4, pp. 2880–2904, Feb. 2022, doi: 10.1021/ACS.JMEDCHEM.1C00598/SUPPL_FILE/JM1C00598_SI_002.CSV.

[63] S. A. Elseginy *et al.*, 'Promising anti-SARS-CoV-2 drugs by effective dual targeting against the viral and host proteases', *Bioorg Med Chem Lett*, vol. 43, p. 128099, Jul. 2021, doi: 10.1016/J.BMCL.2021.128099.

[64] A. Luttens *et al.*, 'Ultralarge Virtual Screening Identifies SARS-CoV-2 Main Protease Inhibitors with Broad-Spectrum Activity against Coronaviruses', *J Am Chem Soc*, vol. 144, no. 7, pp. 2905–2920, Feb. 2022, doi: 10.1021/JACS.1C08402/ASSET/IMAGES/LARGE/JA1C08402_0004.JPEG.

[65] G. G. Rossetti *et al.*, 'Non-covalent SARS-CoV-2 Mpro inhibitors developed from in silico screen hits', *Scientific Reports 2022 12:1*, vol. 12, no. 1, pp. 1–9, Feb. 2022, doi: 10.1038/s41598-022-06306-4.

[66] K. Zhang *et al.*, 'Discovery of quinazolin-4-one-based non-covalent inhibitors targeting the severe acute respiratory syndrome coronavirus 2 main protease (SARS-CoV-2 Mpro)', *Eur J Med Chem*, vol. 257, p. 115487, Sep. 2023, doi: 10.1016/j.ejmech.2023.115487.

[67] A. Citarella *et al.*, 'Discovery of a Novel Trifluoromethyl Diazirine Inhibitor of SARS-CoV-2 Mpro', *Molecules*, vol. 28, no. 2, Jan. 2023, doi: 10.3390/MOLECULES28020514.

[68] H. A. Alhadrami *et al.*, 'Neoechinulin A as a Promising SARS-CoV-2 Mpro Inhibitor: In Vitro and In Silico Study Showing the Ability of Simulations in Discerning Active from Inactive Enzyme Inhibitors', *Mar Drugs*, vol. 20, no. 3, p. 163, Mar. 2022, doi: 10.3390/MD20030163/S1.

[69] Y. Zhang *et al.*, 'Structure-Based Discovery and Structural Basis of a Novel Broad-Spectrum Natural Product against the Main Protease of Coronavirus', *J Virol*, vol. 96, no. 1, pp. 1253–1274, Jan. 2022, doi: 10.1128/JVI.01253-21/SUPPL_FILE/JVI.01253-21-S0001.PDF.

[70] J. W. Zhang *et al.*, 'Discovery of 9,10-dihydrophenanthrene derivatives as SARS-CoV-2 3CLpro inhibitors for treating COVID-19', *Eur J Med Chem*, vol. 228, p. 114030, Jan. 2022, doi: 10.1016/J.EJMECH.2021.114030.

[71] S. K. Samrat *et al.*, 'Allosteric inhibitors of the main protease of SARS-CoV-2', *Antiviral Res*, vol. 205, p. 105381, Sep. 2022, doi: 10.1016/j.antiviral.2022.105381.

[72] O. A. Chaves *et al.*, 'Apixaban, an orally available anticoagulant, inhibits SARS-CoV-2 replication and its major protease in a non-competitive way', *J Mol Cell Biol*, vol. 14, no. 6, Aug. 2022, doi: 10.1093/jmcb/mjac039.
